# Disparities in mothers’ healthcare seeking behavior for common childhood morbidities in Ethiopia: based on nationally representative data

**DOI:** 10.1186/s12913-021-06704-w

**Published:** 2021-07-08

**Authors:** Nigatu Regassa Geda, Cindy Xin Feng, Susan J. Whiting, Rein Lepnurm, Carol J. Henry, Bonnie Janzen

**Affiliations:** 1 Center for Population Studies, College of Development Studies, Addis Ababa University, Sidist Kilo Campus, PO Box 1176, Addis Ababa, Ethiopia; 2 Department of Community Health and Epidemiology, Faculty of Medicine, Dalhousie University, Halifax, NS Canada; 3 College of Pharmacy and Nutrition, Health Sciences A-Wing, University of Saskatchewan, 107 Wiggins Road, Saskatoon, SK S7N 5E5 Canada; 4 School of Public Health, Health Science E-wing, University of Saskatchewan, 104 Clinic Place, Saskatoon, SK S7N 2Z4 Canada; 5 Department of Community Health and Epidemiology, College of Medicine, University of Saskatchewan, Saskatoon, Canada

**Keywords:** Acute respiratory illness, Diarrhea, Determinants, Health services, Morbidity, Ethiopia

## Abstract

**Background:**

Childhood morbidities such as diarrhea and pneumonia are the leading causes of death in Ethiopia. Appropriate healthcare-seeking behavior of mothers for common childhood illnesses could prevent a significant number of these early deaths; however, little nation-wide research has been conducted in Ethiopia to assess mothers’ healthcare-seeking behavior for their under five children.

**Methods:**

The study used the Ethiopian Demographic and Health Surveys (EDHS) data. The EDHS is a cross sectional survey conducted in 2016 on a nationally representative sample of 10,641 respondents. The main determinants of care-seeking during diarrhea and acute respiratory infection (ARI) episodes were assessed using multiple logistic regression analyses while adjusting for complex survey design.

**Results:**

Only 43% and 35% of households sought medical attention for their children in episodes of diarrhea and ARI, respectively, during a reference period of 2 weeks before the survey. The odds of seeking care for diarrhea are lower for non-working mothers versus working mothers. The likelihood of seeking care for diarrhea or ARI is higher for literate fathers compared to those with no education. The place of delivery for the child, receiving postnatal checkup and getting at least one immunization in the past determined the likelihood of seeking care for ARI, but not for diarrhea. The odds of seeking care are higher for both diarrhea and ARI among households that are headed by females and where mothers experienced Intimate Partner Violence (IPV) violence. Religion and types of family structure are also significant factors of seeking care for diarrhea episodes, but not for ARI.

**Conclusions:**

The findings call for more coordinated efforts to ensure equitable access to health care services focusing on mothers living in deprived household environment. Strengthening partnerships with public facilities, private health care practitioners, and community-based organizations in rural areas would help further improve access to the services.

## Background

Reducing preventable deaths of newborns and children under-5 years of age is one of the priority areas of sustainable development [1]. The direct causes of most early age mortality are diseases that are preventable and treatable, namely pneumonia, diarrhea, malaria, and measles (in descending order) [2–4]. Despite efforts in ensuring universal access to health care through the national Health Sector Development Program [5], Ethiopia is still experiencing one of the highest prevalence of poor health outcomes for children, especially regarding under-5 mortality [6]. For children under-5 years old, mortality is 114 deaths per 1,000 live births in rural areas and 83 deaths per 1000 live births in urban areas. Common childhood illnesses and nutritional deficiencies have been the underlying cause for a significant proportion (at least 28%) of all child deaths in Ethiopia [7]. Most of these lives could be saved through affordable treatment measures like antibiotics for acute respiratory infections, oral rehydration for diarrheal diseases, and the use of appropriate drugs for malaria [1, 8]. Poor healthcare-seeking behavior of parents has been shown to contribute to ineffective prevention and control of morbidity and mortality related to health conditions [9].

In this regard, research-based evidence on parents’ care-seeking behavior related to common morbidities is required in order to design appropriate child survival strategies in countries like Ethiopia, where the early mortality rate is high [10]. The most recent data indicate that close to 10% of children under-5 experienced episode of diarrhea or acute respiratory infection (ARI) during the last 15 days prior to the survey date [6]. Only a small proportion of children with common childhood illnesses receive appropriate health care [8, 10]. While care-seeking behavior is generally influenced by availability, quality of services, and personal choices, the roles played by maternal and household variables are crucial. Low care-seeking behavior is particularly pronounced among households in the poorest quintile, in rural areas, with poor parental education, and those who are non-users of basic maternal and child health services [8, 10–12]. Other contributing factors could be: accessibility to service; severity of illness; trust in healthcare providers; and prior beliefs concerning treatment of the illness [13].

Previous studies conducted on the health of children in Ethiopia have focused on small/ local areas [8, 10] using micro-level data or have addressed only one population category (such as rural areas) [14]. Also, most of these local studies did not adequately address the contribution of certain maternal and household determinants, such as the role of intimate partner’s violence, type of family structure, religion and access to health facilities. The present study addresses these limitations by using nationally representative data and considers a wide range of individual and contextual variables. Drawing from the existing literature, it is hypothesized that care-seeking from public/private health facilities during episodes of the two most common childhood illnesses, namely diarrhea and ARI would be lower among those who did not have access to key health services for children especially antenatal care (ANC), institutional delivery service, postnatal care, and basic immunization services. It is also posited that poor care-seeking would be more a function of maternal factors such as the living context, literacy, and resources of these women.

Therefore, this study aims to assess the disparities in mothers’ healthcare-seeking behavior for common childhood morbidities (diarrhea and ARI). Given the high morbidity and mortality rates for under-5 children in Ethiopia, a deeper understanding of the health-seeking behavior of mothers would help health program planning and monitoring.

## Methods

### Conceptual framework

**Fig. 1:**
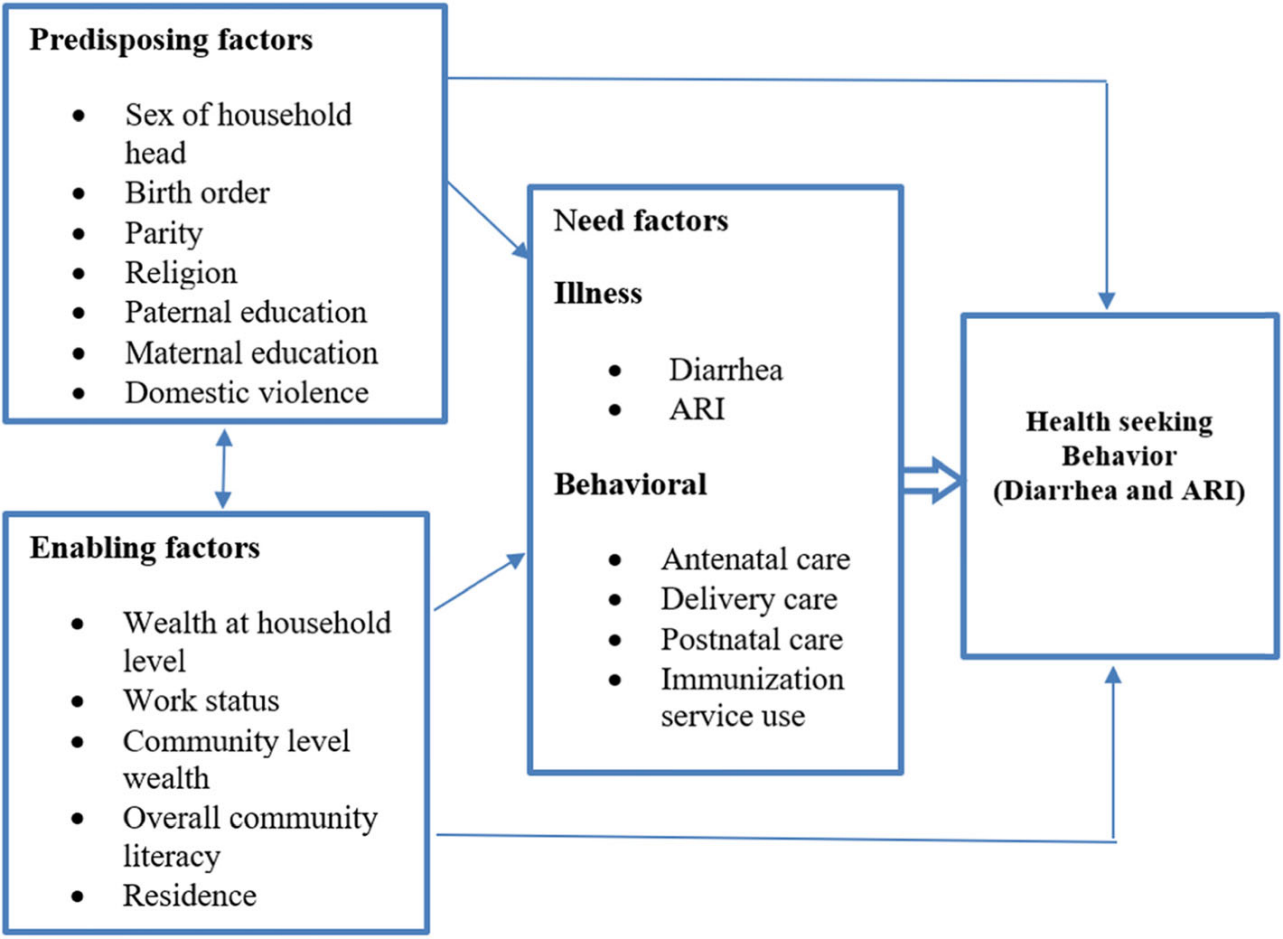
Conceptual framework of the study developed based on Anderson’s behavioral model of health service utilization [15]

The conceptual framework (Fig. 1) for factors of health seeking behaviors for mothers of children with diarrhea and ARI conditions is based on Anderson's behavioral model [15]. The model assumes that health-seeking behavior is a function of three sets of characteristics: predisposing, enabling, and need. The actual seeking of health services is assumed to be a sequential and conditional function of the individual’s predisposition to use health services, their perceived need to use them, and their ability to obtain the services [5].

### Data and study population

The present study was conducted using a nationally representative data of the most recent Ethiopian Demographic and Health Survey (EDHS, 2016). The data were collected from 10,641 ever-married women (15–49 years), who had given birth in the last three years prior to the survey.

### Study design, sampling, and data collection procedures

The EDHS is a cross-sectional study which collected demographic and socioeconomic data at a specific point in the life of the respondents. It employed a two-stage stratified cluster sampling [6]. The first stage of sampling selected 645 enumeration areas (EAs) randomly from all administrative regions. In the second stage, households, within the selected EAs,were drawn using systematic random sampling [6]. For the present analysis, only those who reported episodes of ARI (*n* = 1280) and diarrhea illness (*n* = 1227) during the two weeks preceding the survey date were considered.

The EDHS data collection used a standardized questionnaire, that has been used in more than 100 countries. Information on child’s experience of diarrhea/ARI episodes and care seeking was collected from closest caregiver of the child, mostly the biological mother. The data collection followed a standard procedure with adequate field staff training, pretesting, filed supervision and data quality maintenance. The detailed description of methods, design, instruments, participants, and sampling frame have been published by the Central Statistics Authority of Ethiopia and Macro International [6].

### Ethical consideration

Permission to use the data for the purposes of the present study was granted by ORC Macro International and Central Statistics Authority. Ethical approval for this study was obtained from the University of Saskatchewan, Canada.

### Measures of outcome and exposure variables

Two dichotomous outcome variables were considered in the current analysis: appropriate seeking of treatment for diarrhea and ARI, respectively, constructed based on mothers’ responses to questions on recent episodes of various forms of morbidities. Appropriate healthcare-seeking behavior was defined as situations when mothers visited any health facility/institution during episodes of childhood illnesses for diarrhea or ARI. Information on the diarrhea episode during the reference period of two weeks was used. Diarrhea is described as an abnormal increase in the frequency, volume, or liquidity of stools, lasting from a few hours to several days [16, 17]. ARI is derived based on mothers' responses to questions if their child had a fever, cough, chest congestion, or short rapid breaths in the two weeks preceding the survey [6]. The WHO referred these symptoms as “suspected pneumonia” [6]. The EDHS survey considered them as a proxy measure of pneumonia [16]. In a follow-up question, mothers who reported the occurrence of these symptoms were asked if the child required medical attention for any episode during the reference period. Health care seeking behavior was coded as “1” if mothers sought care during such episodes and “0” if no care was sought.

The choice of potential explanatory variables was guided by literature reviews and model fitting procedures. To capture the key determinants of care-seeking, a wide range of explanatory variables were included. For the purpose of this analysis, the explanatory variables were divided into three major categories: (1) Predisposing factors (sex of household head, birth order, parity, religion, paternal education, maternal education, Intimate Partner Violence (IPV)); (2) enabling factors (wealth at household level, work status, community-level wealth status, overall community level education and residence); and (3) need factors includes behavioral variables (utilization of ANC, delivery care, postnatal care, and immunization). Most of the background variables such as child’s sex, age, parental literacy status, type of family structure, parity was used the way they were coded in the original data. The remaining variables were constructed by combining certain items. Of importance are those measured using indices such as IPV, household wealth index, community-level wealth, and community-level maternal education. IPV was constructed from mother’s binary response for five sets of questions about her experiences of violence by her partner (beating, insulting, causing physical assault, chasing from home, and slapping). The variable was dichotomized into no IPV and at least one IPV experience during a reference period of one year prior to the survey. Household wealth was estimated in the EDHS with an asset-based index that combined information about ownership of consumer goods, housing quality, and water and sanitation facilities [6]. This is a combined measure of the cumulative living standard. Community-level wealth was measured based on the mean of the wealth index of each household in a cluster. Similarly, mean maternal education at the community level was measured based on information on the highest education achieved by each individual woman. These two variables were used in the analysis as continuous variables.

### Statistical analysis

Data were analyzed using SPSS, version 20 [18]. Percentage and frequency were used to describe the distribution of child morbidity status and care-seeking by selected socio-demographic characteristics. Multicollinearity among the explanatory variables was checked using the Variance Inflation Factor (VIF). Bivariate logistic regression was conducted to select the variables with p-values < 0.2. Multiple logistic regression analyses were then conducted to examine the association between selected explanatory variables and the two-health care seeking variables. Both crude odds ratio (COR) and adjusted odds ratio (AOR) with 95% confidence interval (CI) were computed. A backward model selection method was employed. We used a *p*-value ≤ 0.05 to ascertain statistical significance [19]. Two-way interactions were assessed by entering the product of two hypothesized variables. The final multivariable model contained only statistically significant variables. All analyses were weighted per DHS guideline [6].

## Results

The analysis was conducted on the reported cases of 1227 diarrhea and 1280 ARI. The proportion of households representing urban and rural residents was 11% and 89%, respectively (Table 1). Many of the respondents (50.7%) reported household sizes of 4–6 followed by households of 7 or more (38.8%). The average household size for the reported cases was 6.02 ± 2.28. The distribution of respondents by asset-based wealth status showed a difference of about 9.5% between the top and bottom quantiles. About 11% of the respondents were engaged in polygamous marriages; they were in their prime reproductive ages (20–34) with a median age of 19.2 years. There was high disparity in the proportion of literacy status of mothers and fathers. Only 34% of mothers were literate compared to 49% for fathers. A total of 1227 (11.8%) mothers reported at least one under-5 child with a diarrhea episode in the two weeks preceding the survey, and a total of 1280 (11.3%) mothers reported at least one under-5 child with an ARI episode.

**Table 1:** Descriptive statistics of mothers’ care-seeking for their children’s diarrhea and ARI episodes during the 2 weeks preceding the survey date in Ethiopia, 2016

Characteristics	n (%)	Diarrhea (n = 1227)	ARI (n = 1280)
		Yes (%)	Yes (%)
Sex of the household head			
Male	9494 (86.1)	449 (41.3)	283 (24.8)
Female	1529 (13.9)	75 (52.8)	66 (47.1)
Residence			
Urban	1216 (11.0)	74 (58.7)	50 (53.8)
Rural	9807 (89.0)	450 (40.9)	299 (25.2)
Family structure			
Monogamous	9243 (83.9)	451 (43.5)	283 (26.4)
Polygamous	1219 (11.1)	42 (35.3)	40 (25.6)
Place of delivery			
Institution	1216 (11.0)	200 (52.2)	155 (42.9)
Home	9807 (89.0)	324 (38.3)	194 (21.1)
Religion			
Orthodox Christians	3772 (34.2)	203 (45.0)	138 (29.2)
Other	7251 (65.8)	321 (41.3)	210 (26.0)
Mothers work status			
Working	2988 (27.1)	181 (48.4)	135 (34.7)
Not working	8035 (72.9)	343 (40.2)	214 (24.0)
Postnatal check			
No postnatal check for most recent birth	9706 (88.1)	98 (50.0)	79 (42.9)
Had post-natal check for most recent birth	1316 (11.9)	426 (41.3)	270 (24.6)
Intimate partners violence			
No	2763 (25.1)	112 (38.6)	65 (19.1)
Yes, at least one type of violence	8259 (74.9)	412 (44.0)	284 (30.2)
Children ever born			
1–3	4836 (43.9)	292 (48.8)	172 (30.8)
4–6	3732 (33.9)	155 (36.6)	107 (24.7)
6 +	2454 (22.2)	77 (37.4)	70 (24.3)
Birth order			
First	2058 (18.7)	110 (44.0)	93 (36.0)
Other	8965 (81.3)	413 (42.3)	255 (25.0)
Household wealth			
Poorer and poorest	5156 (46.8)	200 (37.2)	129 (23.1)
Medium	2280 (20.7)	103 (38.4)	75 (24.0)
Richer and richest	3587 (32.5)	221 (52.4)	144 (35.2)
Immunization			
Had at least one vaccination	9616 (87.2)	477 (43.4)	326 (29.1)
Never had vaccination	1407 (12.8)	47 (36.4)	22 (13.8)
Mothers’ literacy status			
Literate	3739 (33.9)	352 (38.8	247 (24.7)
Illiterate	7284 (66.1)	172 (53.9)	102 (36.4)
Fathers’ literacy status			
Literate	5385 (48.9)	290 (44.0)	209 (30.8)
Illiterate	5637 (51.1)	234 (41.1)	140 (23.3)

Tables 2 and 3 present the results of the bivariate (unadjusted effects) and multivariable (adjusted effects) analysis of care-seeking for diarrhea episodes and ARI, respectively. The odds of diarrheal care-seeking decrease by 33% for non-working mothers compared to those working outside the home (AOR = 0.672, 95% CI 0.453–0.996). Fathers’ literacy was significant in both models, where the likelihood of care-seeking increases when fathers have some education. Child’s place of delivery, receiving postnatal checkup and getting at least one immunization services in the past determined the likelihood of care-seeking for ARI episodes, but not for diarrhea. For instance, mothers who delivered their child at home have a lower chance of taking their ill child to medical attention in case of ARI episodes compared to those who were born in health facilities (AOR = 0.551, 95% CI 0.392–0.773). The odds of care-seeking for ARI episodes decrease by about 44% for those who never had postnatal checkup (AOR = 0.548, 95% CI 0.358–0.839) compared to those mothers who had a postnatal checkup. Mothers whose children did not receive immunization in the past are also less likely to seek care for children’s ARI episodes compared to mothers whose children who had at least one immunization (AOR = 0.475, 95% CI 0.285–0.792).

**Table 2:** Unadjusted and adjusted odds ratio for determinants of mothers’ treatment-seeking behavior for diarrhea episode in Ethiopia, 2016 (n = 1227)

*Characteristics*	Unadjusted		Adjusted (backward elimination) *	
	COR (95% Cl)	p-value	AOR (95% CI)	p-value
Sex of the household head				
Male				
Female	0.726 (1.081–2.758)	0.022	2.182 (1.143–4.164)	0.018
Family structure				
Monogamous				
Polygamous	0.447 (.243–0.824)	0.010	0.381 (0.188–0.770)	0.007
Religion				
Orthodox				
Other	0.616 (.440–0.864)	0.005	0.545 (0.367–0.808)	0.003
Women work status				
Working				
Not working	0.642 (.454–0.909)	0.012	0.672 (0.453–0.996)	0.048
Intimate partners violence				
No				
Yes (at least one type of violence)	2.086 (1.378–3.157)	0.001	1.826 (1.152–2.893)	0.010
Father’s literacy status				
Literate				
Illiterate	0.679 (0.486–0.949)	0.023	0.629 (0.417–0.949)	0.027
Household wealth				
Poorer and poorest				
Medium	1.785 (1.161–2.743)	0.008		
Richer and richest	1.562 (1.072–2.277)	0.020		
Place of delivery				
Institution				
Home	0.604 (0.429–0.851)	0.004		
Postnatal check				
Yes				
No	0.570 (0.369–0.881)	0.011		
Children ever born				
1–3				
4–6	0.574 (0.393–0.839)	0.004		
6 +	0.678 (0.431–1.065)	0.092		
Birth order				
First				
Other	0.773 (1.527–1.133)	0.187		
Immunization				
Had at least one vaccination				
Never had vaccination	0.359 (0.196–0.660)	0.001		
Mothers’ literacy status				
Literate				
Illiterate	0.562 (0.403–0.784)	0.001		
Residence				
Urban				
Rural	0.566 (0.337–0.949)	0.031		
Wealth at community level (%)	1.285 (1.075–1.536)	0.006		
Women education at the community level (mean years of schooling completed)	1.224 (1.100–1.363)	0.000	1.192 (1.055–1.347)	0.005

*The logistic regression for adjusted AOR was computed using backward elimination method which provides outputs only for significant (p-value < 0.05) variables

**Table 3:** Unadjusted and adjusted odds ratio for determinants of mothers’ treatment-seeking behavior for ARI episode in Ethiopia, 2016 (*n* = 1280)

Characteristics	Unadjusted		Adjusted (backward elimination) *	
	COR (95% Cl)	*p*-value	AOR (95% CI)	*p*-value
Sex of the household head				
Male				
Female	2.286 (1.569–3.331)	0.000	1.861 (1.233–2.808)	0.003
Place of delivery				
Institution				
Home	0.341 (0.258–0.452)	0.000	0.551 (0.392–0.773)	0.001
Postnatal check-up				
Yes				
No	0.332 (0.230–0.480)	0.000	0.548 (0.358–0.839)	0.006
Intimate partners violence				
No				
Yes, low to high	1.998 (1.457–2.740)	0.000	1.556 (1.106–2.190)	0.011
Birth order				
First				
Other	0.522 (0.381–0.716)	0.000	0.641 (0.450–0.911)	0.013
Immunization				
Had at least one vaccination				
Never had vaccination	0.457 (0.281–0.742)	0.000	0.475 (0.285–0.792)	0.004
Father’s literacy status				
Literate				
Illiterate	1.537 (1.167–2.024)	0.002	0.672 (0.504–0.896)	0.007
Household wealth				
Poorer and poorest				
Medium	0.951 (0.678–1.334)	0.772		
Richer and richest	1.970 (1.456–2.665)	0.000		
Type of family structure				
Monogamous				
Polygamous	1.141 (0.839–1.550)	0.401		
Mother’s literacy status				
Literate				
Illiterate	1.537 (1.167–2.024)	0.000		
Women work status				
Working				
Not working	0.644 (0.490–0.847)	0.002		
Children ever born				
1–3				
4–6	0.739 (0.549–0.995)	0.046		
6 +	0.787 (0.558–1.109)	0.171		
Residence				
Urban				
Rural	0.246 (0.150–0.404)	0.000		
Wealth at community level (%)	1.478 (1.283–0.703)	0.000		
Women education at the community level (mean years of schooling completed)	1.191 (1.098–0.292)	0.000		

*The logistic regression for adjusted AOR was computed using backward elimination method which provides outputs only for significant (p-value < 0.05) variables

Among the household and community variables, the odds of care-seeking increase in both diarrhea and ARI for female-headed households compared to male-headed households (AOR = 2.182, 95% CI 1.143–4.164 and AOR = 1.861, 95% CI 1.233–2.808, respectively). The likelihood of care-seeking for diarrhea episodes decreases by about 62% in women living in polygamous households compared to those living in monogamous ones (AOR = 0.38, 95% CI 0.0188–0.770). Mothers who experienced low to high domestic violence have increased the likelihood of care-seeking behavior by 1.83 and 1.56 times for their children’s diarrhea and ARI episodes, respectively. Compared to followers of Orthodox Christianity, women from other religious faiths (Muslim, Catholic Christians, and others) have lower odds of care-seeking in case of diarrhea episodes (AOR = 0.545, 95% CI 0.367–0.808).

## Discussion

The study shows that less than half (43%) of households sought medical care for diarrhea and barely over a third (35%) of households sought medical care for symptoms of ARI. Though the low reporting of care-seeking behavior is partly attributed to poor health infrastructure and poverty at the national scale, this study argued that the likelihood of seeking care for diarrhea and ARI is heavily determined by a set of predisposing and enabling factors at the individual level.

Among the predisposing characteristics of children, birth order is the only predictor significant for ARI only. There is an inverse relationship between the birth order of the child and care-seeking during the ARI episode, suggesting that care-seeking declined for the second and subsequent births. The increased confidence mothers develop during subsequent births partly explains why many mothers did not seek medical attention for symptoms of diarrhea and ARI [20]. Interestingly, the analysis indicated significant effects of fathers’ literacy status on mothers’ healthcare seeking for diarrhea and ARI. The effect of fathers’ literacy may stem from the fact that they usually have higher education level compared to mothers. This provides fathers with better opportunities to get access to health information from printed media to understand the health outcomes of childhood illnesses. As can be noted from Table 1, the proportion of literate fathers is much higher (49%) compared to mothers (34%). It is usually the mother’s responsibility to take the child to health facilities. The health-education researchers around the world [21, 22] reported the effect of successful completion of primary schooling or functional literacy as enough to promote child health and survival. A study in India found that even after controlling for assets owned by the household, the probability of seeking care increases with the educational qualification of the father [23]. Although the bivariate analysis showed a significant association between mother’s education and the two outcomes, it turns out insignificant once other variables were included in the models.

The effect of religion on care-seeking behavior agreed with earlier studies. A study in rural Ethiopia reported that Orthodox Christian households are more likely to seek modern health care and seek care earlier compared to Muslim-headed households [14]. Similarly, another local study on maternal health-seeking behavior based on the Ethiopian DHS found that Muslim women are less likely to seek postnatal care compared to Orthodox Christian women [24]. In the present study, mothers from Orthodox Christianity were more likely to seek treatment for diarrhea episodes. The exact path through which religion influences care seeking behavior is not clear. However, it can be hypothesized that difference in the level of literacy, family structure and maternal autonomy between the respondents from Orthodox Christian and Muslim religions might have contributed to this finding. Further analysis of the data for percentage distribution by religion suggests that respondents from Muslim religion had generally lower literacy rate, practice polygamous marital form (which was found having adverse effects on care seeking) and lower autonomy index.

There are few studies conducted to assess the effects of polygamous family structure on health-seeking for childhood morbidities. After controlling for household wealth and maternal education, our result suggests significant adverse effects of mothers in polygamous marriages on care-seeking during diarrhea episodes. One pioneer study on polygamy in Africa reported that being in a polygamous household has little impact on the likelihood of children receiving medical treatment for fever or diarrhea [25]. On the contrary, Arthi and Fenske (2018) reported that polygyny is negatively associated with a range of indicators of early life care in the Nigerian DHS [26]. They also pointed out that competition between wives with the same husband leads to relative inefficiency in resource production and consumption compared to hypothetically more harmonious monogamous unions, in turn reducing child health [26]. In 2012, Henrich, Boyd, and Richerson [27] further reiterated that polygamous men usually prefer to divert their resources into accumulating additional wives rather than into raising existing offspring.

Another striking finding of the present study is the positive association between mothers’ experiences of Intimate Partner Violence and treatment-seeking for both diarrhea and ARI. Consistent with this finding, a recent study in Bangladesh [28] reported that infants of mothers exposed to different forms of family violence had 26–37% higher incidence of diarrhea. In another study in India, treatment-seeking was most prevalent in women who had been exposed to a combination of physical, sexual, and emotional abuse (48.8%) [29]. Given the fact that most Ethiopian women have poor education and low autonomy, it aggravates the likelihood of women experiencing different forms of domestic violence.

The study clearly reiterated that the use of basic maternal and child services (institutional delivery, postnatal care, and basic immunization) makes significant differences in the likelihood of developing care-seeking behavior for ARI but not for diarrhea. Studies in other developing countries reported that fever and ARI were more frequently treated at a facility, while diarrhea was usually treated at first at home [30, 31]. The use of the simple and standard treatment for diarrhea treatment (ORS or HRS) remains sub-optimal in many countries including Ethiopia. In a recent study in Tanzania, for instance, almost all children (99%) treated at home received ORS or HRS) [30]. Some previous studies have indicated the continuum effects of attending ANC and delivery on subsequent use of health facilities. They claimed that these basic health services are commonly used as an opportunity for health promotion [32]. Thus, women who attended ANC/delivery/postnatal care services can easily acclimatize to the health facility environment [33]. This may help them avoid unnecessary fear and stress related to the utilization of childcare and related services. In Ethiopia, the eight basic vaccination services are provided at both the conventional health facilities and through occasional village campaigns. Such campaigns are also usually used to counsel and educate women about signs and symptoms of common childhood illnesses and the risks associated with them.

### Strength and limitations

As the study was conducted based on nationally representative data, the generalizability of the current study to a wider population group is a major strength. Further, the factors analyzed in this study have not been addressed much in previous studies, and hence, sheds light on possible interventions to improve child health and survival in Ethiopia. However, certain limitations warrant careful interpretation of the results of the study. First, care-seeking was examined for only two common childhood morbidities (diarrhea and ARI) due to data limitations. Second, childhood morbidity is season dependent. A longitudinal study may be more suitable to provide data covering different seasons. Third, the DHS system generated the morbidity data based on mothers’ reports of their children's health in the past two weeks preceding the survey. The responses could be biased as they depend on mothers’ perceptions of reality than on clinical examination, and hence, might have introduced some reporting bias and recall bias, creating either under-reporting or over-reporting of childhood illnesses. Due to a lack of data, the present study did not address some of the factors that significantly affect health-seeking behavior such as socio-cultural taboos and prevalence of traditional healthcare in the environment, accessibility to service, the trust in healthcare providers, and prior beliefs concerning the treatment of the illness. There could also be under or over estimation of care seeking for ARI/diarrhea episodes as DHS did not collect data on mothers’ perceptions of the severity of the illness.

## Conclusions

Our study indicated that a substantial proportion of Ethiopian women did not seek health care for their children's diarrheal and AIR conditions. Mothers’ health seeking behavior for common childhood morbidities is determined by a wide range of maternal, household and community variables. More importantly, marital form, experience of IPV, and access to key health services (such as and postnatal care services) appeared to be important determinants of health care seeking in Ethiopia. Based on the findings, more coordinated efforts should be made to ensure equitable access to health care services focusing on mothers living in deprived household environment. Strengthening partnerships with public facilities, private health care practitioners, and community-based organizations would help further improve access to the services. Promoting continuous community-level health education should be more crucial areas of concern for rural health extension workers and program administrators in Ethiopia.

## Data Availability

The datasets used for this study are made available from ICF international/DHS program at https://dhsprogram.com/data/Access-Instructions.cfm. Thus, administrative permissions were required to access the raw data from this organization. Public access to the database is open upon permission.

## Abbreviations

ANC: Antenatal care
ARI: Acute respiratory infection
CSA: Central statistics authority
DHS: Demographic and health surveys (DHS).
EDHS: Ethiopian demographic and health surveys
GDP: Gross domestic product
IPV: Intimate partners violence
SDGs: Sustainable development goals
SES: Socio-economic status
US: United States
WHO: World Health Organization
